# Caregiver‐Driven Cognitive training (CDCT) – A feasible approach to dementia care in the Indian Population

**DOI:** 10.1002/alz.095600

**Published:** 2025-01-09

**Authors:** Aparna Kanmani S, Keshav J Kumar, PT Sivakumar, PS Mathuranath

**Affiliations:** ^1^ National Institute of Mental health and Neuroscience ‐ HOSPITAL, BENGALURU, Karnataka India; ^2^ National Institute of Mental health and Neuroscience ‐ HOSPITAL, BENGALURU, 560029 India; ^3^ Departmet of Psychiatry, National Institute of Mental Health and Neurosciences (NIMHANS), Bengaluru, Karnataka India; ^4^ National Institute of Mental Health and Neurosciences, Bengaluru, Karnataka India

## Abstract

**Background:**

Dementia care is incomplete without active caregiver involvement. Yet, existing cognitive interventions often overlook this crucial aspect. This study aims to develop and evaluate the feasibility of a Caregiver Driven Cognitive Training (CDCT) program in the Indian context, recognizing caregivers as essential partners in the care process.

**Method:**

In Phase I, the CDCT Program was meticulously crafted through a comprehensive literature review and expert input, resulting in nine tailored cognitive tasks spanning attention, executive function, memory, and language domains. Caregivers, whether family members or professionals, were trained as co‐therapists with guidance from neuropsychologists. Phase II involved a pilot test (n = 5), using a quasi‐experimental research design (n = 5) by recruiting a dyad ‐ 1 patient with dementia (with a diagnosis of AD/FTD) + 1 caregiver. Eligible and consenting dyads were allocated (1:1) to the CDCT group or Treatment as usual (TAU) group and received intervention for five weeks. A thus modified CDCT was used in Phase III data collection (n = 32), utilizing the NIMHANS Neuropsychological Battery for Elderly (NNB‐E) and caregiver‐reported scales to assess outcomes assessed at three points: baseline, post‐intervention, and one month follow‐up

**Result:**

The CDCT Program yielded encouraging results, with participants demonstrating improved cognitive function, particularly in attention, executive function, and memory, post‐intervention compared to a Treatment as Usual (TAU) group. Additionally, CDCT participants exhibited enhanced quality of life, increased independence in daily activities, and reduced symptom severity. Caregivers reported subjective improvements in confidence, error detection, self‐correction, communication, and insight. The program received high feasibility ratings from caregivers, indicating its suitability for the Indian population. The quantitative and qualitative evidence of the same shall be presented.

**Conclusion:**

The CDCT program represents a paradigm shift in dementia care, emphasizing the active involvement of caregivers as co‐therapists. This approach not only enhances patient outcomes but also empowers caregivers, fostering a sustainable model of cognitive care tailored to the Indian socio‐cultural context.

